# Mid-pregnancy psychosocial distress and the risk of third-trimester small-for-gestational-age fetuses: a prospective cohort study in coastal Indonesia

**DOI:** 10.3389/fpsyt.2026.1859239

**Published:** 2026-07-31

**Authors:** Suriyanti Suriyanti, Veni Hadju, Ridwan Mochtar Thaha, Yahya Yahya, Anna Khuzaimah, Musfira Musfira

**Affiliations:** 1 Doctoral in Public Health Science, Faculty of Public Health, Hasanuddin University, Makassar, Indonesia; 2 Departement of Nutrition, Faculty of Public Health, Hasanuddin University, Makassar, Indonesia; 3 Departement of Health Promotion, Faculty of Public Health, Hasanuddin University, Makassar, Indonesia; 4 Departement of Anthropology, Faculty of Social and Political Sciences, Hasanuddin University, Makassar, Indonesia

**Keywords:** coastal, distress, fetal growth, maternal, mental health

## Abstract

**Background:**

Evidence regarding the association between second-trimester psychosocial distress and fetal growth remains limited, particularly in coastal populations of low- and middle-income countries. This study aimed to investigate whether psychosocial distress during the second trimester is associated with an increased risk of having a small-for-gestational-age (SGA) fetus in the third trimester.

**Methods:**

A prospective cohort study was conducted among 293 pregnant women in their second trimester across three coastal sub-districts. Psychosocial distress was assessed using the DASS-42, while fetal growth was evaluated through Estimated Fetal Weight (EFW) obtained via ultrasonography. Multivariate Poisson regression with robust standard errors, clustered by primary health care, was employed to evaluate the association between psychosocial distress and SGA.

**Results:**

The median maternal age was 27 years (IQR 22–32); most participants’ husbands worked in the informal sector (94.5%), and the majority came from low-income households (65.2%). Bivariate analysis revealed that second-trimester psychosocial distress (CRR = 4.25; 95% CI: 2.35–7.67; p<0.001) and maternal age (CRR = 2.37; 95% CI: 1.36–4.12; p=0.002) were significantly associated with third-trimester SGA. After adjusting for maternal age and pregnancy complications, these associations remained statistically significant (ARR = 4.00; 95% CI: 2.76–5.80; p<0.01).

**Conclusion:**

Psychosocial distress in the second trimester is significantly associated with the risk of SGA in the third trimester. These findings underscore the importance of integrating mental health screening into routine antenatal care, particularly for populations at heightened social and environmental vulnerability.

## 1 Introduction

Globally, small-for-gestational-age (SGA) infants remain a major public health concern requiring urgent attention. Approximately 17% of infants are born SGA each year, with the majority of cases occurring in low- and middle-income countries (1). SGA is a key indicator of intrauterine growth restriction and is defined as a fetal or birth weight below the 10th percentile for gestational age, according to standardized fetal growth references such as INTERGROWTH-21st (2, 3). SGA is frequently associated with elevated neonatal morbidity and mortality (3, 4) and has long-term adverse consequences, including altered carbohydrate and lipid metabolism (5), impaired motor and cognitive development (5–7), and an increased risk of anemia in children at 6, 12, and 55 months of age (8).

Several studies have examined the biological determinants of SGA, including pregnancy-associated hypertensive disorders (9–11), maternal anemia (12, 13), and nutritional status (14). Placental dysfunction has also been implicated in SGA through mechanisms such as uteroplacental insufficiency (15). However, these biological factors cannot fully explain the occurrence of SGA, particularly among pregnancies without complications, highlighting the need to explore non-biological and modifiable determinants such as psychosocial distress. Psychosocial distress during the second trimester may contribute to SGA risk in the third trimester, yet its influence has been less recognized compared to biological factors (11, 14, 15).

Evidence indicates that maternal psychosocial distress, including symptoms of depression, anxiety, and stress, can affect fetal programming during pregnancy (16) through behavioral and biological pathways that directly influence intrauterine growth (17–19). Nonetheless, evidence specifically linking second-trimester psychosocial distress to third-trimester SGA remains limited. Previous studies measuring stress, anxiety, and depression around 20 weeks of gestation support the second trimester as a critical window for psychological influences on fetal growth (20).

The second trimester represents a crucial phase for the development of fetal physiological systems and placental adaptation (21). It is also the period when the fetus undergoes rapid and active growth, with increases in biometric parameters, linear organ development, and substantial brain volume expansion (21, 22). Psychosocial stress during this phase may disrupt these intensive growth processes and subsequently affect fetal development in the third trimester. Epidemiological studies have consistently linked maternal stress to impaired fetal growth (20, 23).

Fetal growth restriction (SGA) during pregnancy is commonly assessed via Estimated Fetal Weight (EFW) obtained through ultrasonography (24–26). Assessing third-trimester SGA using EFW provides both clinical and epidemiological value, enabling early detection and potential intervention before delivery (27). Cohort studies indicate that fetuses below the 10th percentile in the third trimester face increased risks of adverse pregnancy outcomes and long-term health burdens (28). However, many previous studies rely on birth weight alone as a growth indicator (18, 29), which inadequately captures fetal growth dynamics before birth.

Coastal populations are particularly vulnerable due to low socioeconomic status and environmental stressors (30–32). In such contexts, psychosocial distress may play a more pronounced role in maternal and fetal health. Therefore, this study aimed to investigate whether psychosocial distress during the second trimester is associated with the risk of third-trimester SGA among pregnant women living in coastal areas.

## 2 Methods

### 2.1 Study location and time

This study was conducted in Takalar Regency, a coastal area with a higher population density compared to other sub-districts. The study period spanned approximately six months, from February to August 2025. Primary health care in these three sub-districts was predominantly provided by Community Health Centers, with each sub-district having two facilities offering maternal health services in accordance with the Indonesian Ministry of Health standards.

### 2.2 Study design

A quantitative prospective cohort design was employed to assess whether psychosocial distress in pregnant women during the second trimester was associated with the risk of fetal growth restriction (SGA) as measured by ultrasound-based biometric assessments in the third trimester. Prospective cohort studies are particularly suitable for evaluating temporal relationships between exposure and outcome, allowing for the calculation of incidence and relative risk (33, 34). Participants were stratified into two groups based on their exposure status in the second trimester and were followed prospectively through the third trimester to observe outcomes.

### 2.3 Participants

The study population comprised all pregnant women residing in the coastal areas of Takalar Regency. Inclusion criteria were: second-trimester pregnancy (gestational age 14–27 weeks), residence in one of the three coastal sub-districts, and willingness to participate. Exclusion criteria included pregnant women requiring intensive care and those with multiple pregnancies. Eligible participants underwent semi-structured interviews to assess psychosocial distress during the second trimester and were subsequently followed through the third trimester. Fetal growth was evaluated using EFW obtained via ultrasound at primary health care.

**Figure 1:**
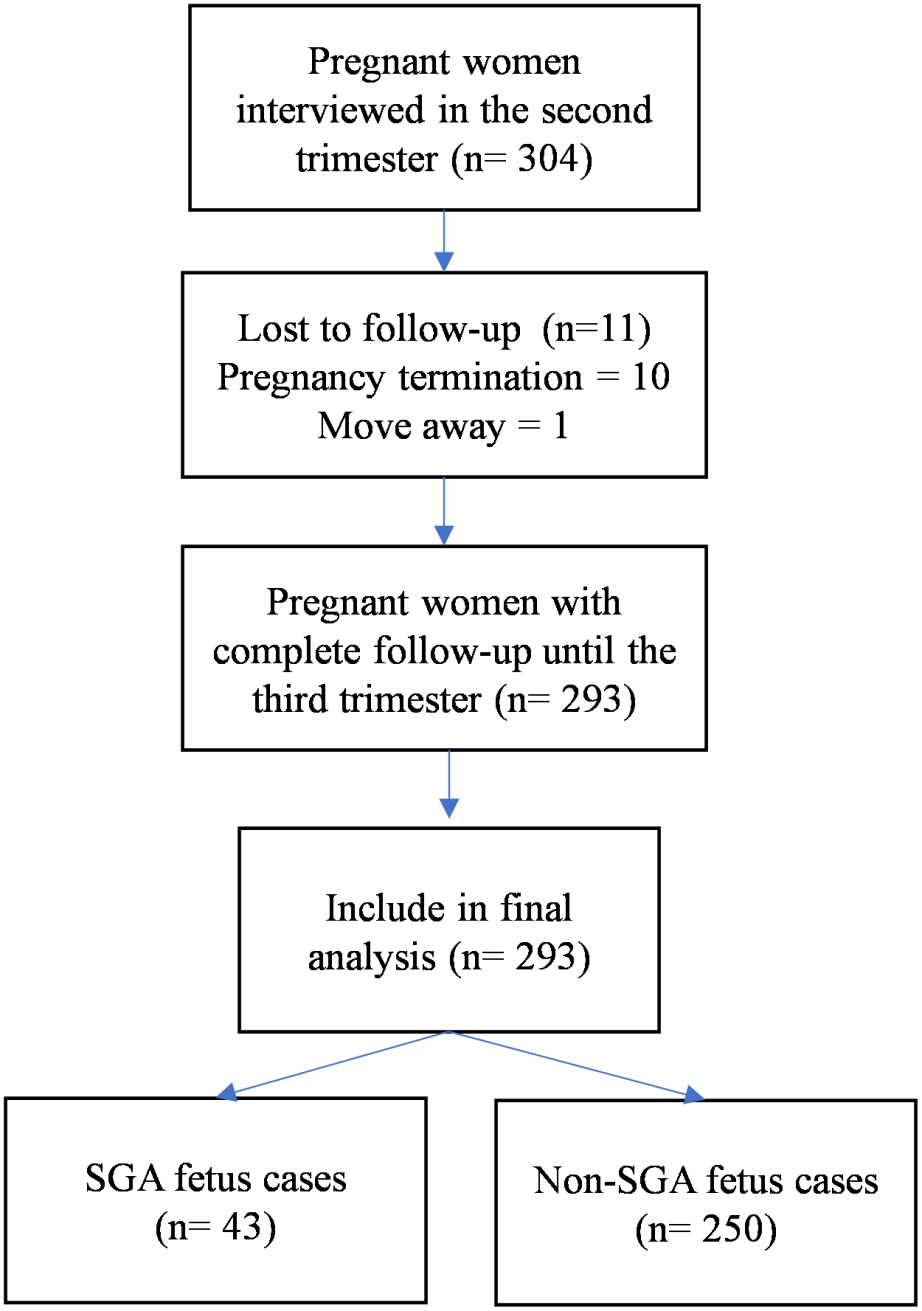
Flow diagram of participant selection.

Participants were recruited sequentially until the minimum sample size of 293 was reached, as determined using the Slovin formula (35). Recruitment involved collaboration with health workers on duty at primary health care, who coordinated with community health cadres to gather eligible pregnant women. Data collectors provided study explanations, obtained informed consent, and conducted psychosocial assessments. Follow-up continued through the third trimester, during which fetal growth outcomes were assessed based on EFW results from ultrasound examinations performed at the health facilities. **Figure 1**.

### 2.4 Data collection

#### 2.4.1 Outcome variables

The primary outcome, SGA, was determined based on EFW in the third trimester, assessed via ultrasonography performed by certified physicians. Data analysis accounted for clustering, and sensitivity analyses were conducted to minimize potential response bias and operator variation. This approach ensured consistent fetal growth estimates, although EFW remains a predictive measure rather than a direct measurement of birth weight. Using EFW to assess SGA provides substantial clinical and epidemiological value, as it enables early detection of intrauterine growth restriction prior to delivery (27). EFW was calculated using the standard Hadlock formula. Gestational age was determined from first-trimester ultrasound, the first day of the last menstrual period (LMP), and the date of the ultrasound examination. EFW values were compared with gestational age using the INTERGROWTH-21st fetal growth standards, and a fetus was classified as SGA if its EFW fell below the 10th percentile for gestational age.

#### 2.4.2 Independent variables

Second-trimester maternal psychosocial distress was assessed using the DASS-42 questionnaire (Lovibond & Lovibond, 1995), which has been internationally validated for reliability and construct validity. The DASS-42 comprises 42 items across three subscales—depression, anxiety, and stress—using a Likert scale ranging from 0 to 3, reflecting participants’ experiences over the past week. Although not specifically developed for pregnant women in coastal areas, the DASS-42 was piloted with 50 pregnant women in Tamalate District, Makassar City, adjacent to the study site, to ensure comprehension. The pilot demonstrated that each subscale was valid and reliable, with Cronbach’s alpha values exceeding 0.6.

Psychosocial distress was defined as the presence of at least mild symptoms in one or more subscales according to standard thresholds. Data were collected through semi-structured interviews conducted by trained enumerators, comprising undergraduate and graduate students from the Faculty of Public Health, guided by an interview protocol developed by the research team. KoBoToolbox software and choice-of-response cards were used to standardize data collection.

#### 2.4.3 Covariate variables

Covariate variables considered in this study included maternal demographic, obstetric, and environmental factors that could potentially confound the association between psychosocial distress and fetal growth. Maternal age was classified as high-risk (<20 years or >35 years) or low-risk (20–35 years). Parity was categorized as nulliparous or parous, including both primiparous and multiparous women. The interpregnancy interval was considered as less than 24 months or 24 months and above. Maternal education was classified as high (completion of high school or equivalent) or low (below high school). Employment status was categorized as formal (structured employment characterized by a fixed salary and a formal employment contract) or informal (employment included self-employment, casual labor, and other forms of work without a formal employment contract or regular fixed income). Household income was defined as the average total monthly income earned by all household members, expressed in Indonesian rupiah (IDR). Based on the income intervals specified in the questionnaire, households were classified into two categories: low income (<IDR 1 million and IDR 1–<2 million per month) and high income (IDR 2–3 million and >IDR 3 million per month). Pregnancy complications, including gestational hypertension, gestational diabetes, anemia, and hyperemesis gravidarum, were recorded as present or absent. Maternal nutritional status was assessed using mid-upper arm circumference (MUAC) to identify Chronic Energy Deficiency. Additional covariates included exposure to domestic violence (yes/no) and secondhand smoke (SHS) exposure within the household (exposed/not exposed). These covariates were included to adjust for potential confounding effects on the association between second-trimester psychosocial distress and third-trimester SGA.

#### 2.4.4 Statistical analysis

Data were analyzed using Stata MP 19.5. Univariate analysis was performed to describe the frequency distribution of all variables. Bivariate analysis employed modified Poisson regression with robust standard errors to estimate crude risk ratios (CRR) and corresponding p-values. Variables with a p-value <0.25 in the bivariate analysis were considered candidates for multivariate analysis.

Multivariate analysis was conducted using modified Poisson regression with robust standard errors, clustered at the primary health care level, to obtain adjusted risk ratios (ARR) with 95% confidence intervals (CI) and p-values. Clustering by primary health care facility accounted for potential intra-cluster correlations. Multicollinearity was assessed using a correlation matrix and the Variance Inflation Factor (VIF). Covariates included in the multivariate model were selected based on theoretical considerations and previous evidence. Variables with p-values <0.025 were initially included, and non-significant variables were sequentially excluded using purposive selection. Strong confounders, defined as those causing a change in the risk ratio of the main exposure >10%, and theoretically important variables were retained. Event-per-variable (EPV) was maintained at ≥10 to ensure model stability.

Finally, a sensitivity analysis using logistic regression was performed to assess the robustness of the final model. This approach ensured that the reported associations between second-trimester psychosocial distress and third-trimester SGA were reliable and accounted for potential confounding and clustering effects.

#### 2.4.5 Ethics approval

This study has obtained approval from the Ethics Committee of the Faculty of Public Health, Hasanuddin University, under protocol number 21325093013 and permit number 665/UN4.14.1/TP.01.02/2025.

## 3 Results

A total of 293 pregnant women in their second trimester participated in this study. As shown in **Table 1**, the median maternal age was 27 years (IQR 22–32). More than half of the participants had a high level of education (54.6%), and the majority were employed in the informal sector (96.9%). Similarly, most participants’ husbands worked in the informal sector (94.5%). A substantial proportion of the participants (65.2%) came from low-income households. The study population was drawn from six community health centers, with Bontomarannu Community Health Center contributing the largest number of participants (19.1%), whereas Aeng Towa Community Health Center contributed the fewest (8.5%).

**Table 1:** Demographic characteristics of pregnant women (n=293).

Characteristics	Value
Maternal age, years	27 (22–32)
Level of education, n (%)	
High	160 (54.6)
Low	133 (45.4)
Maternal employment, n (%)	
Formal	9 (3.1)
Informal	284 (96.9)
Husband’s employment, n (%)	
Formal	16 (9.5)
Informal	277 (94.5)
Household income level, n (%)	
High	102 (34.8)
Low	191 (65.2)
Primary health care, n (%)	
Aeng Towa	53 (18.1)
Bontomangape	25 (8.5)
Bontomarannu	56 (19.1)
Bontokassi	54 (18.4)
Galesong	52 (17.8)
Galesong Utara	53 (18.1)

Continuous variables are presented ad median (interquartile range and categorical variables as n (%).

**Figure 2:**
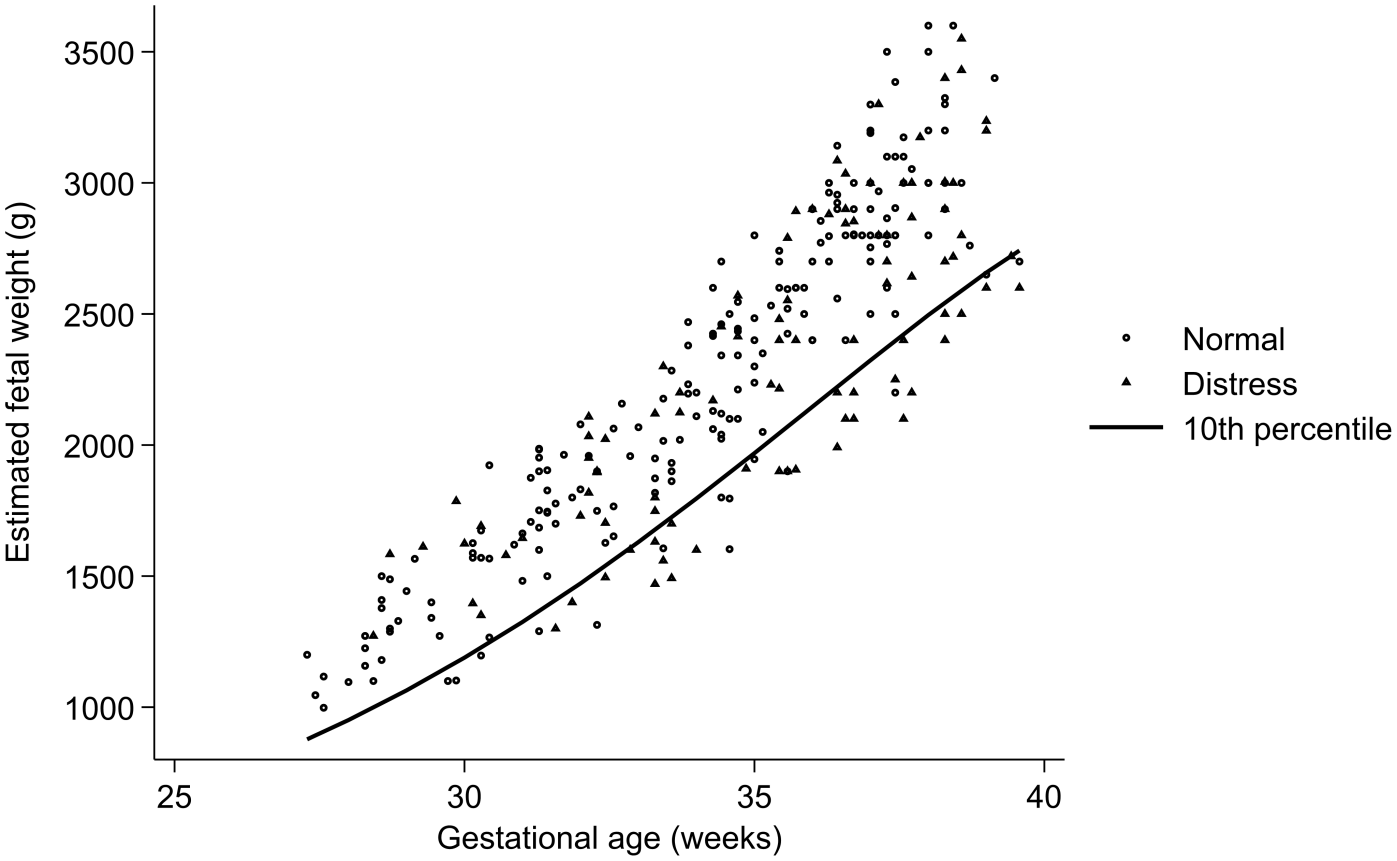
Scatter plot of estimated fetal weight (g) versus gestational age (weeks) with 10^th^ percentile growth curve (cut-off for third-trimester SGA) according to maternal psychosocial distress.

**Figure 2** illustrates a trend toward third-trimester SGA among pregnant women experiencing psychosocial distress, evident from the increasing proportion of data points for the distress group that fall near or below the 10th percentile line.

**Table 2** presents the results of a bivariate analysis conducted using modified Poisson regression with robust standard errors. The findings indicate that pregnant women experiencing psychosocial distress during the second trimester had a significantly associated with an increased risk of having a small-for-gestational-age (SGA) fetus in the third trimester (CRR = 4.25; 95% CI: 2.35–7.67; p < 0.001). Maternal age also demonstrated a significant association with third-trimester SGA (CRR = 2.37; 95% CI: 1.36–4.12; p = 0.002). Variables found to be significant in the bivariate analysis, including psychosocial distress and maternal age, were subsequently included in the multivariate analysis. Additionally, other variables with p-values below 0.25, namely maternal education level (CRR = 1.51; 95% CI: 0.87–2.65; p = 0.141) and pregnancy complications (CRR = 1.66; 95% CI: 0.93–2.95; p = 0.085), were incorporated into the multivariate model for further assessment.

**Table 2:** Bivariate analysis using modified Poisson regression with robust standard errors (n=293).

Variables	SGA n (%)	Non-SGA n (%)	Crude RR	95%CI	P-value
Psychosocial distress					
Distres	29 (30.2)	67 (69.8)	4.25	2.35-7.67	< 0.001
Normal	14 (7.1)	183 (92.9)	1.00	Ref	
Maternal age					
High risk (<20 or >35 years)	15 (27.8)	39 (72.2)	2.37	1.36-4.12	0.002
Low risk (20–35 years)	28 (11.7)	211 (88.2)	1.00	Ref	
Maternal education level					
Low	24 (18.1)	109 (81.9)	1.51	0.87-2.65	0.141
High	19 (11.9)	141 (88.1)	1.00	Ref	
Household income level					
Low	30 (15.7)	161 (84.3)	1.23	0.67-2.25	0.499
High	13 (12.8)	89 (87.3)	1.00	Ref	
Parity					
Parous	27 (13.3)	176 (86.7)	0.74	0.42-1.31	0.316
Nulliparous	16 (17.8)	74 (82.2)	1.00	Ref	
Inter pregnancy interval					
< 24 month	4 (12.5)	28 (87.5)	1.01	0.37-2.74	0.982
≥ 24 month	22 (12.4)	156 (87.6)	1.00	Ref	
Pregnancy complication					
Yes	14 (21.2)	52 (78.79)	1.66	0.93-2.95	0.085
No	29 (12.8)	198 (87.2)	1.00	Ref	
Chronic Energy Deficiency					
Yes	9 (17.6)	42 (82.4)	1.25	0.64-2.45	0.505
No	34 (14.0)	208 (86.0)	1.00	Ref	

*Variables with bivariate analysis results of p<0.25 were included in multivariate analysis using a purposeful selection approach.

**Table 3** presents the findings of a multivariate analysis performed using modified Poisson regression with robust standard errors clustered at the primary health care level. The final model indicated a strong association between psychosocial distress during the second trimester and an increased risk with third-trimester SGA, after adjusting for maternal age and pregnancy complications. Pregnant women experiencing psychosocial distress in the second trimester had a fourfold higher risk of third-trimester SGA (ARR = 4.00; 95% CI: 2.76–5.80; p < 0.01). Moreover, women in the high-risk age groups (<20 years or >35 years) also exhibited a statistically significant association with third-trimester SGA (ARR = 2.03; 95% CI: 1.53–2.70; p < 0.01). Although pregnancy complications were associated with an increased risk of SGA, this relationship did not reach statistical significance (ARR = 1.42; 95% CI: 0.95–2.14; p = 0.085).

**Table 3:** Crude and adjusted risk ratios for third-trimester SGA (n = 293).

Variables	Crude RR (95% CI)	p-value	Adjusted RR (95% CI)	P-value
Psychosocial Distress	4.25 (2.35-7.67)	< 0.001	4.00 (2.76-5.80)	< 0.001
High risk maternal age (<20 or >35 years)	2.37 (1.36-4.12)	0.002	2.03 (1.53-2.70)	<0.001
Any Pregnancy complication	1.66 (0.93-2.95)	0.085	1.42 (0.95-2.14)	0.085

*Crude RR, unadjusted risk ratio; Adjusted RR estimated using Poisson regression with robust standard errors clustered at primary health care; the multivariate mode adjusted for maternal age and pregnancy complication as covariates.

Model adequacy was evaluated using deviance and Pearson goodness-of-fit statistics, with deviance/df = 0.48 and Pearson/df = 0.90, indicating a satisfactory fit to the study data. A sensitivity analysis was performed by rerunning the model without adjusting for primary health care clusters, maternal age, or pregnancy complications. The results demonstrated robust and consistent findings across different model specifications, with the ARR for psychosocial distress ranging from 4.00 to 4.25.

**Figure 3:**
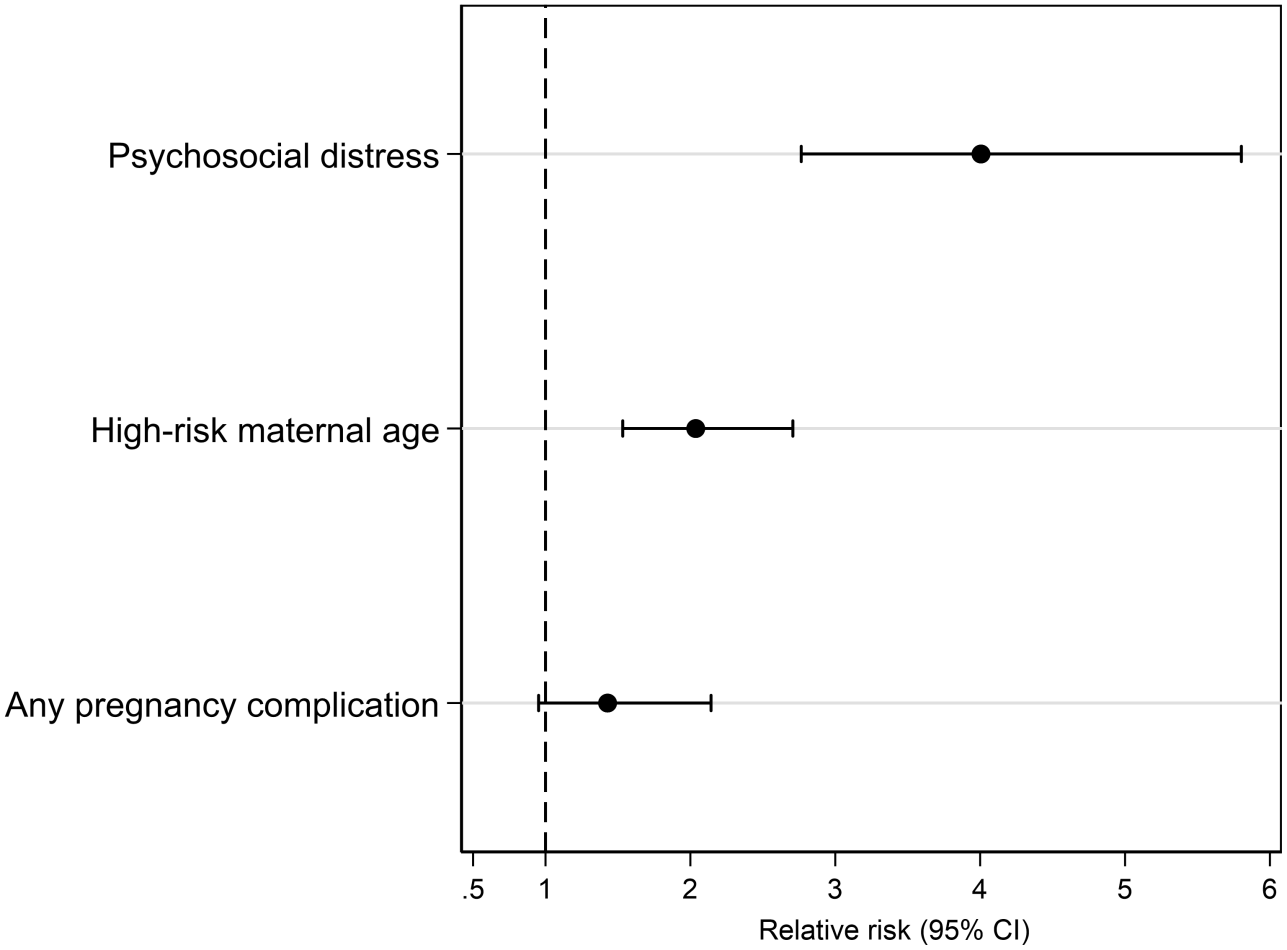
Adjusted relative risks for factors associated with third-trimester SGA.

**Figure 3** illustrates the adjusted relative risk estimates for factors associated with third-trimester SGA outcomes. Psychosocial stress experienced by pregnant women during the second trimester remained strongly associated with an increased risk of third-trimester SGA, even after adjusting for maternal age and pregnancy complications.

## 4 Discussion

This study found that approximately 14.7% of pregnant women in their third trimester in coastal areas carried fetuses classified as SGA. This prevalence underscores a substantial burden of fetal growth restriction in the third trimester, particularly in low- and middle-income countries. The observed prevalence aligns with previous studies reporting SGA rates exceeding 10% of all live births (13, 14, 36). Psychosocial distress experienced during the second trimester was strongly associated with the risk of third-trimester SGA. The CRR was 4.25, and the ARR was 4.00, indicating that covariate adjustment had only a minimal effect on the magnitude of the association. This finding is consistent with existing evidence showing that psychosocial distress is relatively prevalent among pregnant women and may contribute to adverse pregnancy outcomes (37). Prior research has also reported associations between maternal stress even before conception and the occurrence of SGA in newborns (29). In this study, women experiencing psychosocial distress in the second trimester had a fourfold higher risk of having SGA fetus in the third trimester compared with women without such distress, after adjusting for maternal age and pregnancy complications.

Despite these findings, causal interpretations should be approached with caution. The observed association may be influenced by residual confounding factors that were not measured, including maternal nutritional status, environmental exposures, pre-pregnancy mental health conditions, sleep quality, social support, and access to antenatal care. Previous cohort studies also suggest a link between prenatal distress and increased SGA risk, although the underlying causal mechanisms require further validation through rigorous prospective designs with comprehensive confounder control (29). A meta-analysis similarly indicates that prenatal distress increases SGA risk, although the effect is moderate and heterogeneous across populations (38). Consequently, psychosocial distress should be interpreted as an important risk indicator rather than definitive evidence of causality.

This study’s findings are consistent with prior research demonstrating that concurrent maternal anxiety and depression elevate the risk of impaired fetal growth (39). However, the magnitude of the observed risk in this study appears higher compared with international cohort data. For instance, the SCOPE study, which included 5,606 nulliparous women, reported that stress, anxiety, and depression at approximately 20 weeks of gestation increased SGA risk with adjusted odds ratios ranging from 1.14 to 1.56 (20). Similarly, a population-based study in China found that maternal stress prior to pregnancy was associated with an increased SGA risk with an adjusted odds ratio of 1.35 (29).

The elevated risk observed in the coastal population in this study is likely influenced by multiple local factors, including suboptimal maternal nutritional status, higher levels of socioeconomic stress, and limited access to quality prenatal care, such as routine ultrasonography (2, 40, 41). Additionally, environmental exposures specific to coastal areas such as high-salt diets and marine pollution may contribute to impaired fetal growth. A meta-analysis has demonstrated associations between social determinants, nutrition, and prenatal care with the risk of SGA (42). These findings underscore the need for context-specific SGA prevention strategies, including enhanced fetal growth screening, maternal nutrition education, and community-based interventions to mitigate socioeconomic risk factors (2).

Variations in outcomes may also arise from differences in measurement methods. Although ultrasonography enables early detection of fetuses at risk for SGA and is a safe, non-invasive tool for both mother and fetus, third-trimester EFW based SGA has inherent limitations. Operator variability, instrument calibration, and image quality can introduce measurement errors, meaning that EFW does not always correspond precisely to actual birth weight (24, 43, 44). Even when measurements are performed by certified clinicians and adjusted for cluster effects with sensitivity analyses, these potential discrepancies must be considered in interpreting results.

Several biological mechanisms may explain the association between maternal psychosocial stress in the second trimester and SGA in the third trimester. Maternal psychosocial stress can activate the sympathetic nervous system and trigger the release of corticotropin-releasing hormone (CRH) and adrenocorticotropic hormone (ACTH), stimulating cortisol production. Elevated maternal cortisol can cross the placenta, influencing fetal cortisol synthesis and altering fetal programming (18). Furthermore, stress-induced changes in maternal behavior such as reduced nutritional intake, disrupted sleep patterns, and non-adherence to health recommendations can negatively impact fetal growth (45, 46). These findings are consistent with prior research identifying depression, anxiety, and stress is associated with fetal growth status.

In the multivariate analysis, maternal age and pregnancy complications were included as covariates. Women in extreme age groups (<20 years or >35 years) exhibited an increased risk of SGA in the third trimester. These results align with prior studies suggesting that maternal age at conception is associated with SGA, although it is not a dominant determinant (14, 29). Maternal age below 20 years may increase SGA risk due to ongoing maternal growth requiring adequate nutrition and immature reproductive system development. Conversely, maternal age above 35 years is associated with placental dysfunction and a higher likelihood of pregnancy complications (2). While pregnancy complications were linked to a higher risk of SGA, this association did not reach statistical significance after adjustment. Nevertheless, the direction of influence suggests that complications may still contribute to third-trimester SGA risk.

## 5 Limitations and directions for further study

This study has several limitations. First, psychosocial distress was assessed using the self-report DASS-42 instrument, which may introduce response biases, including social desirability and differences in question interpretation. This underscores the need to develop a screening tool better suited for similar populations. Second, unmeasured residual confounding factors such as maternal nutritional status, environmental exposures, pre-pregnancy mental health, sleep quality, social support, and access to antenatal care may have influenced the findings. Third, amniotic fluid assessment was not consistently available in the study dataset. Given that abnormal amniotic fluid volume is clinically associated with placental dysfunction and fetal growth restriction, the lack of this information may have limited the ability to distinguish isolated low estimated fetal weight from cases associated with underlying uteroplacental insufficiency. Fourth, the use of third-trimester EFW remains susceptible to operator variability, instrument calibration, and image quality. Although measurements were performed by certified physicians and adjusted for cluster effects with sensitivity analyses, EFW may not fully reflect actual birth weight. Future research should incorporate additional assessment methods or longitudinal monitoring to improve the accuracy of fetal growth evaluation.

All studies included in this analysis were observational. While the temporal sequence of exposures and outcomes is clear, causal inferences should be made with caution. Nevertheless, these studies provide robust scientific evidence linking second-trimester psychosocial distress to the incidence of third-trimester SGA. The prospective cohort design allows for temporal evaluation of exposures prior to outcomes, providing a basis for subsequent intervention-based research. Moreover, the findings can inform the development of preventive strategies aimed at improving maternal and child health in vulnerable populations during critical periods of gestation.

## 6 Conclusion

Psychosocial distress is associated with fetal growth status in the third trimester. In this prospective cohort study, women experiencing psychosocial distress in the second trimester had more than a fourfold increased risk of having a SGA fetus in the third trimester. These findings underscore the importance of routinely integrating mental health services into maternal healthcare. Furthermore, they highlight the second trimester as a critical period of pregnancy that should not be overlooked, even though it is generally considered more stable than the first and third trimesters.

## Data Availability

The raw data supporting the conclusions of this article will be made available by the authors, without undue reservation.
